# Medical students’ challenges and suggestions regarding research training: a synthesis of comments from a cross- sectional survey

**Published:** 2019-07-24

**Authors:** John J. Riva, Radwa Elsharawi, Julian Daza, Augustin Toma, Robert Whyte, Gina Agarwal, Jason W. Busse

**Affiliations:** 1 Department of Family Medicine, McMaster University, Ontario, Canada; 2 Department of Health Research Methods, Evidence & Impact, McMaster University, Ontario, Canada; 3 Wayne State School of Medicine, Michigan, USA; 4 Michael G. DeGroote School of Medicine, McMaster University, Ontario, Canada; 5 Department of Anesthesia, McMaster University, Ontario, Canada; 6 The Michael G. DeGroote Institute for Pain Research and Care, McMaster University, Ontario, Canada; 7 The Michael G. DeGroote Centre for Medicinal Cannabis Research, McMaster University, Ontario, Canada

## Abstract

**Background:**

We previously reported on a cross-sectional study of students from the Michael G. DeGroote School of Medicine at McMaster University that found most respondents wanted more opportunities to participate in research. Students provided additional comments that we synthesized to enrich the findings of our quantitative analysis.

**Methods:**

From our previously administered 13-item, online questionnaire, run across three campuses in Ontario, Canada, 498 of 618 medical students completed our survey and 360 (72%) provided optional written comments, which we synthesized using thematic analysis in this current study.

**Results:**

Major themes that emerged were: (1) Active student participation to identify research opportunities and interested mentors are needed; (2) Types of research involvement; (3) Uncertainty whether research training translates into useable skills; (4) Desire for a formalized research curriculum and centralization of research opportunities across campuses.

**Conclusion:**

Programs should stress to interested students the importance of actively looking for research opportunities and consider both large and small-group educational sessions.

## Introduction

Medical students often witness curious clinical results, which may prompt some to pursue research to formally explore their experiences. This opportunity to advance knowledge within the greater healthcare system is compelling. Exposure to research experiences during training may improve analytical reasoning, communication skills, and application of emerging knowledge to patient care.^1^ While many areas of medical curricula advance, research education and participation remains less formalized and variable across programs.^2^ In prior studies, medical students have endorsed both the motivation and positive experiences with the formation of their research abilities during their training and highlighted competing demands on their time and limited opportunities as barriers.^3,4^

We previously surveyed all students enrolled in the Michael G. DeGroote School of Medicine, across three distributed campuses, at McMaster University. The aim of the original study was to examine student research interest and participation as well as self- rated research ability among medical students. Most (445 of 498; 89%) respondents had had prior research experiences. While some (159 of 498; 32%) were currently participating in research, most (383 of 498; 86%) wanted more opportunities. In our adjusted logistic regression model, higher rating of supervisors’ research understanding was associated with higher student interest in research (OR=2.1; 95%CI: 1.3-3.4). Also, in our adjusted linear model prior student research work (e.g., thesis) was associated with higher self-rated research abilities. Our survey included an option to provide written comments. We reviewed and synthesized these comments to supplement our previously reported quantitative findings for the purpose of describing the challenges medical students face in participating in research training, as well as their suggestions for how such programs may be improved.

## Methods

The methods for survey development, administration, and quantitative analyses have been reported previously.^5^ In brief, stakeholder consultations and literature informed the development of a 13-item questionnaire that was administered online in 2014, with two follow-up reminders, across three campuses in Hamilton, Niagara, and Kitchener-Waterloo, Ontario, Canada. The current analysis was restricted to written comments provided in four survey questions (see Appendix A). We tested for responder bias by looking for differences in the distributions between responders and non-responders by year in the program and campus using Pearson’s Chi-Square test (χ^2^).

For our thematic analysis, four reviewers developed a preliminary coding system to categorize themes and sub-themes after a discussion around a sample of 10 surveys, using a previously established approach.^6^ Three teams of reviewers then applied this system, independently and in duplicate, to written comments in other surveys until coding became stable, as evidenced by no new codes and disagreement among reviewers being minimal. Teams of reviewers then applied the final coding strategy to all written comments, with each team coding 120 surveys. Each respondent that provided written comments could contribute to more than one theme or sub-theme; however, each theme or sub-theme was only coded once in a single survey to address the issue of clustering.

## Results

There was an overall response rate of 81% to our survey (498 of 618 students), and 360 respondents provided written comment questions to at least one of the four optional questions, for a total of 967 written comments. There was no difference in the distribution of responders and non-responders to written questions by campus (χ^2^ = 4.22; p=0.121) or by year in the program (χ^2^ = 2.25; p=0.522) (see Appendix B). Our coding revealed 4 distinct themes and 28 sub-themes (see Appendix A):

### Theme I. Active student participation and mentorship opportunities are needed (n=265)

Students felt that they needed to actively seek out opportunities to participate in research through talking to faculty or staff, searching the Internet, or joining interest groups. However, they also felt that some researchers were unwilling to take on students with limited research experience. Some students felt the best strategy was to join existing studies and research teams, while others felt it might be easier to initiate their own project. Others were unsure how to approach the search for opportunities and felt that availability of protected time and training in research methodology would be helpful.

### Theme II. Type of research involvement (n=230)

A number of students (77 of 230; 33%) reported no engagement in research at all. Competing demands may limit participation in research. While not specifically asked, a few students (28 of 230; 12%) additionally reported on the types of research methods used in their projects. Half of the students (14 of 28) identified completing either case reports or systematic reviews while the remaining described involvement in observational studies, randomized control trials, quality improvement, or qualitative research.

### Theme III. Uncertainty whether research training translates into useable skills (n=267)

Many 2^nd^ and 3^rd^ year students reported exposure to lectures on clinical epidemiology in large group sessions (65 of 267; 24%), and opportunities to apply this learning by critically appraising research studies in small-group sessions (90 of 267; 34%). Students also recognized the structure in place with respect to education on the use of the library, evidence-based medicine, epidemiology, and scholarship competencies. Although, the perceived practicality of this education and available time to focus on research was unclear to some students; moreover, some questioned whether they would be able to incorporate these skills into clinical practice.

### Theme IV. Desire for a formalized research curriculum and centralization of opportunities (n=205)

Respondents acknowledged that not all medical students were interested in research training, but a number (71 of 205; 35%) felt that increased formalization of research training in their curriculum would be helpful. Specific strategies advanced included formalized testing, keeping a record of previous research activities, and a mandatory formal research project. Others thought that significant curriculum changes, such as offering a summer break or an academic credit for research completed, might improve engagement in research activities by students. Additionally, there was practical direction by some students (49 of 205; 24%) to raise awareness, for example, through the creation of an online portal to facilitate linking interested students with faculty researchers. Lastly, there was also endorsement for an online repository of materials relevant to research training.

## Discussion

Written comments by McMaster medical students highlighted challenges associated with securing research-training opportunities, particularly if they lacked research skills. As a result, some interested students had been unsuccessful in linking up with a research mentor. There is a need to address barriers to medical students identifying research mentors, as successful mentorship is associated with personal development, research productivity, as well as publication and grant success.^7-9^

Other students questioned whether the skills they were acquiring regarding research methodology and critical appraisal of the literature would be practical to apply in clinical practice, which aligns with previous literature suggesting that students perceive a separation between evidence-based medicine and the realities of clinical practice.^4^

Some students suggested greater formalization of research training, which differs from a previous review that suggested curriculum formalization is no different than electives from the perspective of student satisfaction.^1^ Other reviews support greater formalization of research education into medical curricula. Specifically, using research education as a basis for evidence-based medicine, increasing opportunities for students to participate in research, and formalized incorporation of research methodology education into curriculum were found to increase medical students’ participation in research.^10-12^ A sub-theme in our sample that warrants further research is the concept of offering academic credit for research.

We found that students support an online repository to centralize research opportunities. There is increasing inclusion of Internet and telephone technologies in distributed medical education, such as large real-time video-conference displays and classroom interactions between distributed campuses.^13,14^ An increased and applied use of technologies may be important with respect to student engagement around research education and opportunities and recent changes to the Michael G. DeGroote school of medicine curriculum include new online materials summarizing research opportunities for students. Other recent strategies to encourage student research are involvement of students in research-related committees (e.g., training development, journal interest group) and formal review of student research projects.^5^ Further research is needed to evaluate strategies aimed at reducing barriers to student’s participation in research.

### Strengths and limitations

Strengths of our study include coding all written comments independently and in duplicate with one active medical student included in each pair to maintain relevancy with the current program. Our study also has limitations. We coded all unique themes and sub-themes from each survey, which means that some respondents contributed more content to our analysis than others. Approximately 40% of respondents did not provide written comments, and a higher number of 1st year students answered the survey relative to later years, which suggests our findings may be affected by selection bias. For example, 1^st^ year students may be less familiar with the research curriculum and opportunities. Lastly, the generalizability of our findings to other medical programs, particularly without distributed campuses and four-year programs, is uncertain.

### Conclusion

Themes that emerged from this study provide areas of opportunity for medical programs to engage with students, ideally through technologies and mentorship, to improve their research education and opportunities. Programs should stress to interested students the need to be actively looking for research opportunities and consider students’ desire for more formalized large and small-group educational sessions.

## Appendix A

**Summary of Categories for Themes and Sub-Themes Ranked by Frequency & Representative Quotes**

**Question 1:** How would you go about getting more involved in research during the MD program if you were interested?

Theme I: Active student participation and mentorship opportunities are needed Sub-Themes (n=265)	Frequency (%)
Talk to people (administrative staff, research coordinators, classmates, upper years students, faculty supervisors, study PIs)	171 (64.5)
Use various communication methods (e-mail, cold calling, “luck”, in-person meetings, Medportal, Google, interest groups, join a research team)	53 (20.0)
Look for ongoing research (find projects already in progress, continue on with their own current research projects)	50 (18.9)
Suggestions on: [1] facilitators (campus integration, protected time, central source for opportunities; more curriculum) & [2] barriers (no time in medical school; not interested in research)	28 (10.6)
Unsure how to get involved	23 (8.7)
Would self-start their own research project	14 (5.3)
Would take further research training (classes, individual sessions, electives)	10 (2.7)

Representative Quote:

*I have no background in research so I am unsure of the best ways to go about getting involved, but I have been told that the best way to go about this is to contact a physician or researcher who is researching a field that you are interested in and ask them if they are willing to take medical students. This has so far been unsuccessful, and I feel that my lack of prior experience is the major problem. [1**^st^**year student]*

**Question 2:** Please list the topics of any research you currently are undertaking:

Theme II: *Types of research involvement* Sub-Themes (n=230)	Frequency (%)
Not doing any research	77 (33.5)
Systems-based research (basic biological, immune, gastrointestinal, cardiology, endocrine, neurological, hematology, urology, ENT)	54 (23.5)
Medical education (communication. social media, e-health, quality improvement)	42 (18.3)
Secondary Care and Emergency Medicine (trauma, surgery, radiology, cancer)	39 (17.0)
Primary Care (obstetrics, family, geriatrics, palliative)	38 (16.5)
Psychosocial (pain, addiction, mental health, disability) & lifestyle (prevention, obesity, exercise, nutrition, CAM, sports)	29 (12.6)
Reported a research methods used (case-report, observational, trial, systematic review)	28 (12.2)
Local population health (infection, public health, poverty)	15 (6.5)
Global health (health systems and policy)	08 (3.5)

Representative Quote:

*It's difficult without summers as many PI require dedicated time in blocks. I'm not entirely sure how I can go about this. Maybe I can get lucky throughout my MD training by coming across an opportunity where I can get involved in a project without compromising too much time away from MD training. [1**^st^**year student]*

**Question 3:** What particular classes/units in the MD program have provided you education on research methods concepts and translating research in practice?

Theme III: Uncertainty whether research training translates into useable skills Sub-Themes (n=267)	Frequency (%)
Required small-group sessions (preceptors, medical foundations, clinical skills sessions)	90 (33.7)
Pursuing optional research education (medical decision-making online videos, self-study, journal clubs, quality improvement initiatives in program)	76 (28.5)
None (just started medical school, unsure)	71 (26.6)
Required large-group sessions (professional competencies, EBM sessions, epidemiology sessions, library training)	65 (24.3)
Perceived usefulness of current required education (positive and negative perceptions, amount of time relative to whole curriculum, practicality of research education sessions)	13 (4.9)
Culture of training (research cross-cuts training, EBM focus)	07 (2.6)
Clerkship rotations (specifically emergency, internal, nephrology)	04 (1.5)

Representative Quotes:

*During Emergency Medicine Core rotation and Medical Selective, I was asked to critically appraise an article, then present. There were several presentations prior to clerkship, during which epidemiologists presented upon the concepts of clinical epidemiology. However, those sessions were not reinforced well and served more to provide exposure than to develop skills. [3**^rd^**year student]*

*The longitudinal epidemiology sessions have provided the sole basis for structured research training in the context of the MD program. [Problem-based learning] provides a framework to develop a data-gathering and analytical skill set, but there is a paucity of readily-available opportunities to apply scientific principles and research methods to clinically relevant questions. [2**^nd^**year student]*

**Question 4:** Please share with us any thoughts you had on ways to improve the MD program in providing education on research and facilitating research opportunities:

Theme IV: Desire for a formalized research curriculum and centralization of opportunities Sub-Themes (n=205)	Frequency (%)
Suggestions on teaching method and content (topics, amount of time spent, self-help options, online learning ideas, presentation formats)	83 (40.5)
Suggestions on equal opportunity for research participation (central Medportal resource/database, across campuses, formal connection process with researchers, reduce “luck”, desire for more opportunities)	81 (39.5)
Suggestions on better integrating research into curriculum (mandatory large and small-group, curriculum, have formal testing, formal projects, horizontal and block electives)	81 (39.5)
Suggestions to increase awareness of ongoing research (fairs, advertisement, build into class content, teach preceptors to discuss, keep a history of previous projects)	49 (23.9)
Suggestions to improve support for research (staff and faculty mentors, longer summer break to allow for research, offer academic CV credit for research, funding, improve Medportal research section)	33 (16.1)

Representative Quotes:

*Formalize it more. Optional videos and self-tests do not seem sufficient to me, especially given the diverse non-science background of many Mac Med students. Interpreting research correctly is one of the most important skills we will need to have as clinicians. [3**^rd^**year student]*

*Having a centralized database, website, or Medportal webpage with available research opportunities, or a compendium on researchers willing to take on an MD student as a research assistant. [1**^st^**year student]*

## Appendix B

**Summary of Responders and Non-Responders by Year in Program and Campus ***

**Table:** What is your current year in the MD program?

	Year 1	Year 2	Year 3	MD/PhD	Total	
non-responders	52 37.7	52 37.7	30 21.7	4 2.9	138 100.0	n %
Responders	125 39.6	104 32.9	82 26.0	5 1.6	316 100.0	n %
Total	177 39.0	156 34.4	112 24.7	9 2.0	454 100.0	n %
*Pearson chi2(3) = 2.2523 Pr = 0.522*						

**Table:** What is your home base campus?

	Kitchener-Waterloo	Niagara	Hamilton	Total	
non-responders	13 9.42	18 13.04	107 77.54	138 100	n %
responders	44 13.92	57 18.04	215 68.04	316 100	n %
Total	57 12.56	75 16.52	322 70.93	454 100	n %
*Pearson chi2(2) = 4.2240 Pr = 0.121*					

* By year in program of respondents, 39% were first year students, 34% second year, 25% final year and 2% were from the MD/PhD program. Main (Hamilton) campus represented 71% of the respondents, while distributed campuses represented 16% (Niagara) and 13% (Kitchener-Waterloo) respectively. Considering the overall enrolment of 618 in the program, the proportion of potential respondents by campus was similar with main (Hamilton) campus (72% of potential students) versus Niagara and Kitchener- Waterloo campuses (both 14% of potential students respectively).
